# Molecular Mechanism and Potential Targets for Blocking HPV-Induced Lesion Development

**DOI:** 10.1155/2012/278312

**Published:** 2011-12-19

**Authors:** E. Guzmán-Olea, V. H. Bermúdez-Morales, O. Peralta-Zaragoza, K. Torres-Poveda, V. Madrid-Marina

**Affiliations:** ^1^Division of Chronic Infections and Cancer, Research Center for Infectious Diseases, Instituto Nacional de Salud Pública, Avenida Universidad No. 655, Cuernavaca 62100, Morelos, Mexico; ^2^Universidad Politécnica del Estado de Morelos, Boulevard Cuauhnáhuac 566, Jiutepec 62550, Morelos, Mexico

## Abstract

Persistent infection with high-risk HPV is the etiologic agent associated with the development of cervical cancer (CC) development. However, environmental, social, epidemiological, genetic, and host factors may have a joint influence on the risk of disease progression. Cervical lesions caused by HPV infection can be removed naturally by the host immune response and only a small percentage may progress to cancer; thus, the immune response is essential for the control of precursor lesions and CC. We present a review of recent research on the molecular mechanisms that allow HPV-infected cells to evade immune surveillance and potential targets of molecular therapy to inhibit tumor immune escape.

## 1. Introduction

Infection with oncogenic types of human papillomavirus (HPV) is the main etiologic factor in cervical cancer (CC) and in its precursor, neoplasia. CC can be a model system for studying the interactions between cells transformed by an oncogenic agent and the immune system, during the progression of the squamous intraepithelial lesion (SIL) [1].

The majority of women clear HPV infection spontaneously by the host immune response, but persistence of HPV infection has been suggested to be associated with development of SIL [2]. The fact that only a small proportion of HPV-infected individuals will eventually develop cancer of the cervix and the long latency period between primary infections and cancer emergence suggest that additional factors are involved in the progression. Other factors such as genetic susceptibility or alteration of the immune response increase the incidence of HPV-associated lesions. A substantial majority of SILs and cancers develop within a specific region of the cervix, the transformation zone, implying that other exogenous or endogenous factors specific to the anatomical milieu may be conducive to SIL and cancer development [3].

A great number of tumors have been identified in humans, most of them growing after the reproductive age. Somatic mutations allow some antigenic tumors to evade the immune response, to grow successfully and persist in our organism in spite of a functionally adequate immune system. The immunology of tumors associated with infectious agents is a remnant of immune response against external pathogens, and low levels of infectious agents can coexist with T-cell-mediated immunity; as a result, the immune system is unable to eliminate all infected cells [4]. 

In this paper, we review the mechanisms that allow CC cells to evade immune surveillance and the molecular therapy to inhibit tumor immune escape. The tumor immune escape refers to the mechanism by which the body maximizes immune tolerance, through the production of soluble immunosuppressive factors (Interleukin (IL)-10, transforming growth factor-beta1 (TGF-*β*1), tumor-infiltrating cells such as macrophages and granulocytes), and the recruitment of suppressive cells of the adaptive and innate immune system.

## 2. Cervical Cancer Molecular Mechanism

### 2.1. Cellular Immune Response in Cervical Cancer

Tumor immunity in CC is activated by helper T cell type 1 (Th1) cytokines and inhibited by Th2 cytokines. Several cytokines have been shown to contribute to the initiation or suppression of cellular immune responses, such as IL-4, IL-12, IL-10, and/or TGF-*β*1, produced by various cell types, including macrophages, dendritic cells, and keratinocytes [5].

As an approach to understanding the factors involved in the generation and maintenance of an efficient antitumor response in CC, several research groups have examined the local expression profile of Th1, Th2, and Th3 cytokines in HPV-positive CC biopsies. The data indicate that more than 80% of the tumors expressed low levels of CD4 mRNA, with all of them expressing higher CD8 mRNA levels. Most tumors expressed IL-4 and IL-10 messenger RNA (mRNAs) and, most importantly, all of them expressed TGF-*β*1 and interferon *γ* (IFN-*γ*) mRNA. None of the studied tumors expressed IL-12, IL-6, or tumor necrosis factor (TNF) mRNA [5–15]. 

There are more tumor infiltrating T lymphocytes (TIL) in the stroma than in epithelium, in biopsies from women with SIL (including a carcinoma *in situ* and a normal region), and in advanced stages of the disease where CD8+ T cells prevailed [7]. Consistent with other reports [8], it was found that CD8+ T cells are predominant, compared to CD4+ T cells, in women with CC. However, what are the mechanisms behind this distribution as well as behind the inability of these CD8+ T cells to eliminate the tumor in CC remains unclear [7].

Immunohistochemical analysis identified IL-10 only in tumor cells and koilocytic cells, but not in tumor-infiltrating lymphocytes, suggesting that IL-10-producing cells are those transformed by HPV. It was found a correlation between immunostaining for IL-10 protein and the level of IL-10 mRNA expression and supernatants from HPV-transformed cell lines containing IL-10 and TGF-*β*1. These findings show a predominant expression of immunosuppressive cytokines, which help to downregulate tumor-specific immune responses in the tumor microenvironment [6, 10]. Furthermore, using an experimental murine model, it has been demonstrated that HPV16 tumors are not only infiltrated by large numbers of M2-like macrophages (TAM), but there is an expansion of myeloid cells in the spleen and altered T/B lymphocyte ratio in the peripheral lymph nodes of tumor bearing mice, indicating systemic effects initiated by the tumors. It has been shown that TAM and myeloid populations in the spleen of HPV16 tumor-bearing mice are important for tumor growth via stimulation of specific regulatory T cells, in a mechanism partially dependent on IL-10 expression by TAM [16, 17]. Furthermore, in C3 tumor-bearing mice, Gr-1(+) cells completely blocked T-cell response to a peptide presented by major histocompatibility complex class I (MHC class I) *in vitro* and *in vivo*. Blocking of the specific MHC class I molecules on the surface of Gr-1(+) cells completely abrogated the observed effects of these cells. Thus, immature myeloid cells specifically inhibited CD8-mediated Ag-specific T-cell response, but not CD4-mediated T-cell response [18]. This represents a mechanism of cellular immune response inhibition, as a potential therapeutic alternative. 

Previous studies have suggested that a reduced T cell function can be associated with alterations in CD3*ζ* protein expression [8]. Thus, the CD3*ζ* mRNA expression by T cells has been examined as an indicator of possibly decreased T-cell function in CC patients. CC progression has been associated with lower CD3*ζ* mRNA, which was even lower in TIL. Studies were done to determine whether decreased CD3*ζ* mRNA expression correlated with low T-cell proliferation in CC. As expected, there was a significant correlation between low T-cell proliferation and decreased CD3*ζ* mRNA expression by anti-CD3 stimulated T cells. Thus, decreased T-cell function appears to correlate with CC progression, which is in agreement with a decreased T-cell proliferation in CC patients [7].

To establish the possible association of cytokines with levels of CD3*ζ* expression, we evaluated the relationship between a number of cytokines and CD3*ζ* expression by PBL and PBL versus TIL. As expected, there were significant positive associations between CD3*ζ*/IL-2 and CD3*ζ*/IFN-*γ* mRNA expression. Moreover, there was an inverse association for IL-10/CD3*ζ* mRNA expression in PBL. These results show that an optimal expression of CD3*ζ* is associated with expression of IL-2 and IFN-*γ* [7]. Furthermore, it has been demonstrated that *in vivo* suppression of CD3*ζ* chains in patients with CIN can be the result of a circulating factor [15]. We believe that this circulating factor is composed of IL-10 and TGF-*β*1 that reduce CD3*ζ* expression [7]. 

### 2.2. Tumor Immune Evasion in Cervical Cancer

Persistent infection is a prerequisite, but may not be sufficient for progressing to CC. HPV “stealth” and immune evasive mechanisms enable infection to persist [19]. Several conditions are required to establish HPV infection among others: the viral lifecycle occurs within the epithelium, there is no viremia, no cell death, and no inflammation, and a local immunosuppression caused by HPV proteins is present. This immunosuppressive state is characterized by repression of TLR9 signal pathways by E6/E7, influence on IFN-*γ* expression by E6/E7, influence on interferon-dependent signal pathways by E6/E7, induction of TGF-*β*1 expression by E6/E7, induction of IL-10 expression by E2 protein, and reduction of migration of Langerhans cells (reduction of E-cadherin by E6) [20–22]. It has been suggested that to optimize immunotherapy strategies, correction of immune-activating signals, eradication of inhibitory factors, and the evasion of newly developed immunoresistant tumor phenotypes need to be simultaneously considered [23].

## 3. Potential Targets for Blocking HPV-Induced Lesion Development

### 3.1. HPV E2 Protein as a Potential Therapeutic Target

The E2 protein of papillomavirus is a regulatory protein playing crucial roles during the vegetative viral cycle [24]. In HPV-infected cells, the binding to the LCR is thought to repress HPV gene expression, and E2 contributes to the control of cell proliferation by regulating the expression of E6/E7. However, in cervical carcinomas, the HPV genome often becomes integrated into the host genome, resulting in loss of E2 expression [25]. This leads to increased levels of E6/E7 and, as a consequence, increased cell proliferation and, presumably, increased tumourigenesis. Moreover, HPV E2 protein possesses antiproliferative effects when the HPV E2 gene is re-introduced experimentally into HPV-transformed cells [26–29]. Ectopic expression of E2 from HPV 16, 18, and bovine papillomavirus type 1 induces cell cycle arrest, increases cell senescence, and strongly inhibits cell proliferation and increased apoptosis [26, 30–32]. The induction of G1 growth arrest by HPV 18 E2 protein in HeLa cells it has been associated with the E6/E7 oncogenes repression, which induces stabilization of p53 [29]. In addition, the induction of apoptosis by HPV E2 is shown in many HPV-negative carcinoma cell lines, such as C33 A (cervical cancer), MCF7 (breast cancer), Saos-2 (osteosarcoma). Furthermore, E2-mediated apoptosis is not specific to transformed cells as it also occurs in primary epithelial cells [31, 33, 34]. 

These results show that the proapoptotic activity of E2 is independent of other viral functions, and more specifically of the transcriptional repression of the E6/E7 viral oncogenes. This activity appears as an autonomous function by which the E2 protein can directly modify cell physiology. So the HPV E2 will be a potential therapeutic toll to repress the E6/E7 oncogenes, inducing inhibition of cell proliferation and cell death by apoptosis in cervical cells. 

In addition to its proapoptotic activity, HPV 16 E2 protein possesses antitumor effects in nude mice bearing tumors generated by inoculation of human HPV-transformed cell lines [35, 36]. Immunization of animals with a recombinant vaccinia virus containing the papillomavirus E2 protein promotes tumor regression and decreases the number of new papilloma that are formed [37]. On the other hand, the administration of an adenovirus HPV 16 E2 recombinant had antitumor effects on an experimental tumor in immunocompetent mice inoculated with the BMK-16/myc (murine cell line transformed with HPV16), indicating antitumor effects of the HPV 16 E2 [38]. These results highlight the clinically relevant therapeutic targets derived from the possible use of the HPV E2 protein for the prevention and treatment of HPV-associated cancer. 

### 3.2. Cytokine-Based Therapies in an Immunosuppressive State in Cervical Cancer

The study of immune conditions permissive to tumor regression is a component of a broad strategy aimed at the identification of more effective therapeutic strategies. The cytokines that are produced in the tumour microenvironment have an important role in cancer pathogenesis. Cytokines are released in response to infection; inflammation and immunity can function to inhibit tumour development and progression. Alternatively, cancer cells can respond to host-derived cytokines that promote growth, attenuate apoptosis and facilitate invasion and metastasis. A more detailed understanding of cytokine tumour cell interactions provides new opportunities for improving cancer immunotherapy [39].

In cervical tissue with HPV infection, anti-inflammatory and immunosuppressive cytokines are expressed in the cervical microenvironment, determining the persistence of HPV and tumor progression by subverting cellular immune surveillance mechanisms the shift is a secondary effect induced by the tumor cells, or may be due to the persistence of the viral infection itself [12, 40]. Certain cytokines (IL-4, IL-10, and TGF-*β*) are highly expressed locally in biopsies from patients with premalignant lesions and CC, and may have induced a local immunosuppression state. In particular, IL-10 is highly expressed in tumor cells and its expression is directly proportional to the development of HPV-positive CC, suggesting an important role of HPV proteins in the expression of IL-10 [6, 11]. The elevated expression of IL-10 may allow for virus persistency, the transformation of cervical epithelial cells, and consequently cancer development. These findings may probably point toward the potential usefulness of cytokine assays for determining prognosis or this lack of immune stimulation may be overcome by enhancing the presentation of the tumor antigens to T cells and by delivering immunostimulatory cytokines [41].

Modification of the immune response against cancer, using specific cytokines, may prove effective against cancers such as CC [41]. Of all cytokines tested in several experimental tumor models, IL-2 and IL-12 seem to have the strongest antitumor activity [42]. IL-2 is a stronger stimulator of proliferation and cytolytic activity that induces Th1-type immune responses through inducing the maturation of Th1 cells from uncommitted T-cell population. IL-12 is a stronger inducer of IFN-*γ* from natural killer (NK) cells. Furthermore, IL-12 was able to inhibit angiogenesis through the IFN-inducible protein-10 [43]. Several studies have reported that as a protein, IL-12 has a critical role in inducing antiviral and antitumor effects *in vivo*. Direct administration by gene therapy cDNA expressing IL-12 or IL-12 protein can affect tumor progression and metastasis in animal models [44–46]. In particular in CC, the direct intratumoral injection of adenovirus expressing IL-12 (AdIL-12) resulted in a significant suppression of tumor growth in a CC animal model system. The injection of AdIL-12 with E7 antigen into either a tumor site or the distance site, along with AdIL-12, further enhanced antitumor effects significantly, more than AdIL-12 or E7 protein injection alone [47]. The antitumor effect of IL-12 was associated with enhancement of IFN-*γ* levels and induction of antigen-specific CD8+ T-cell response. In addition, the treatment with IL-12 gene has been employed using nonviral gene therapy (naked DNA), viral gene therapy [48] with the use of adenovirus [47], *ex vivo* gene therapy [49], and in combination with the E6/E7 oncogenes [48–50], as well as genes of immunomodulatory molecules of the cellular immune response such as B7 [49]. Suppression of tumor growth was observed in all cases. IL-12 is able to inhibit experimental metastasis formation and is considered a good candidate for gene therapy against CC [47]. Additionally, IL-12 gene therapy against CC has been used in conjunction with other cytokines such as GM-CSF and IL-2, resulting in an increase of the protective effect against tumor growth [51]. 

Additionally, a contribution to tumour progression in CC, by immunosuppressive cytokines such as IL-10, has been previously suggested [6, 11]. IL-10 is a Th2-type pleiotropic cytokine that is produced at the tumour site and is increased in sera of patients suffering from different cancer types [52]. IL-10 has been shown to hinder a number of immune functions, for example T-lymphocyte proliferation, Th1-type cytokine production, antigen presentation, and lymphokine-activated killer cell cytotoxicity [10, 53]. One of the main actions of this cytokine is its ability to inhibit the production of proinflammatory cytokines, such as TNF-*α*, IL-1, and IL-12, which are synthesized by macrophages in response to bacterial components, such as lipopolysaccharides (LPS) [14]. This activity results in decreased IFN-*γ* production by macrophages and Th1 lymphocytes and inhibition of cell-mediated immune responses, while concomitantly enhancing humoral immunity [50, 54]. Furthermore, IL-10 strongly reduces antigen-specific T-cell proliferation by inhibiting the antigen-presenting capability of monocytes by down-regulation of the expression of their major histocompatibility complex class II (MHC-II) [55]. IL-10 is endowed with multiple positive regulatory activities: it is a growth factor for mature and immature T cells, it enhances the growth and differentiation of CD28+ cytotoxic T lymphocytes (CTLs), and it induces MHC-II expression in resting B cells, sustaining their viability *in vitro* [56–58].

Since IL-10 has potent immunosuppressive and antiinflammatory properties and is produced by some cancers, it has been hypothesized that its production by tumor cells may contribute to the escape from immune surveillance [11]; however, the results obtained in *in vivo* models are controversial.

The increase of tumour growth by IL-10 could be induced by at least three different simultaneous mechanisms: direct stimulation of cell proliferation through an autocrine mechanism, induction of angiogenesis, and the suppression of the local immune system.

Particularly in CC, immunosuppression is the main mechanism proposed by which IL-10 could promote tumour growth in human tumours and murine models [11, 59]. It has been shown that IL-10 can be an autocrine growth factor in culture cells [60]. A previous study reported the effect of IL-10 on tumour growth in a mouse melanoma model and the induction of cell proliferation, either through autocrine stimulation of tumour cells or just through depression of the immune system. To confirm that the enhanced tumour growth was exerted by IL-10 secretion, the tumour growth of the highest IL-10-producer cells (B16-10) was explored in mice treated with an IL-10-neutralizing antibody. In these mice, the tumours grew slower than in control mice and behaved in a similar way to those induced by nontransfected tumour cells, confirming that IL-10 secreted by transfected cells is actually promoting the tumour growth in a melanoma-B16 model. All the effects induced by IL-10 were prevented in mice treated with a neutralizing anti-IL-10 monoclonal antibody. In several *in vitro* models, IL-10 may inhibit different immune mechanisms involved in the antitumour response. However, in mouse models, the effect of IL-10 on the anti-tumour immune response is controversial. In some models, IL-10 inhibits tumoral growth by stimulation of the immune system, mostly of CTLs and NK cells. In other models, the IL-10 promotes the tumoral growth through a local immunosuppression, inhibiting APC functions, CTLs, and the Th1 response. This contradiction might be explained by differences in IL-10-mediated outcomes due to concentration-dependent effects. In CC tumour models where IL-10 suppresses the immune response and increases tumour growth, the production of IL-10 is much lower [61]. It is important to mention that inhibition of IL-10 production by T cells or malignant cells, using low-dose cyclophosphamide [62], anti-IL-10/IL-10R-blocking antibodies [63, 64], or anti-IL-10 antisense oligonucleotides [65], improves cancer-specific immune responses in some preclinical tumor models, which leads the authors to advocate the use of IL-10-neutralizing agents as immunological adjuvants in the design of anticancer vaccines [53]. 

### 3.3. MicroRNAs and Cervical Cancer

Knowledge about the RNA interference (RNAi) mechanism has progressed considerably and now we know that microRNAs are a new family of small endogenous RNA that have diverse sequences, have independent tissue-specific and time-specific expression patterns, are evolutionarily conserved, and are implicated in posttranscriptional regulatory mechanisms for the silencing of the expression of sequence-specific genes [66–68]. We know that most eukaryotic organisms have a great number of genes that are transcribed as small RNA called microRNA, which are natural effector molecules of the RNAi mechanism in eukaryotic cells [69]. MicroRNAs induce their effects at the mRNA level, by arresting the translation or inducing the cleavage of target mRNA. The level at which the specific microRNA and mRNA are complementary defines which process will be carried out. The pathway in which the nucleotides in microRNA and mRNA are perfectly complementary induces cleavage of transcripts, while mismatches between several unpaired bases produce an arrest of translation [69]. Therefore, for the human species, the relevance of gene expression silencing by RNAi will be better understood when we know the molecular components and the regulatory mechanisms of this process, in normal physiological conditions as well as during the development of pathologies that have gene expression disruption, such as in the carcinogenesis process. Many efforts have been made to design new drugs and develop gene therapy to treat CC [41]. Alternatively, it has been demonstrated that the RNAi mechanism represses the expression of viral oncogenes at the posttranscriptional level, by several orders of magnitude and more efficiently than another treatments [70, 71]. Therefore, knowledge of molecular events in gene expression silencing by RNAi, and their applications during CC development, are a real and efficient gene therapy strategy against the development of this neoplasia.

### 3.4. siRNAs for HPV E6 and E7 Oncogenes, as Potential Gene Therapy for Cervical Cancer

RNAi may silence the expression of genes that encode for tumoral antigens or viral oncogenes, in order to repress the specific proliferation of cancerous cells. As a consequence, the silencing of genes by RNAi is a potential mechanism to inactivate foreign DNA sequences and a successful strategy to silence the expression of HPV oncogenes in CC. The findings reported by several groups in this kind of studies are summarized in Table 1. 

The first studies carried out with synthetic siRNA in order to induce the silencing of HPV16 E6 and E7 oncogenes expression were developed by Jiang and Milner in 2002 [72].In this study, the authors showed the biological effect of siRNAs in human cells from cervical carcinoma. The administration of siRNAs led to mRNA cleavage and the specific silencing of HPV16 E6 and E7 oncogenes expression. Besides this, E6 silencing induced the expression of gene p53, transactivation of the inhibiting gene of p21-CIP1/WAF1 cyclin-kinase, and decrease of cellular proliferation, whereas silencing of E7 induced cellular death by apoptosis. Thus, the findings reported by this group demonstrated, for the first time, that the expression of HPV E6 and E7 oncogenes may be specifically silenced by siRNAs in human tumoral cervical cells that have been transformed by HPV.

### 3.5. Silencing of HPV E6 and E7 Bicistron with siRNAs

Attention has been focused on an aspect of the use of siRNAs for HPV E6/E7 oncogenes, which is their ability to silence the HPV E6-E7 bicistron. The effect of synthetic siRNA for HPV16 E6 oncogene on SiHa cells (HPV16+) has been reported and the silencing of both E6 and E7 oncogenes has been observed [73]. In addition, observations have been made as to the inhibition of cellular proliferation, p53 protein expression, the induction of p21-CIP1/WAF1 gene, and the identification of the cell cycle arrest mediator—the hypophosphorylated form of pRb associates—as well as the inactivation of E2F transcription factor. Furthermore, when the SiHa cells were inoculated in immune deficient (SCID) mice and treated with siRNAs for E6 oncogene, a decrease in the ability of cancerous cells to induce tumor formation in the animals was detected. Therefore, the findings reported by this group show that siRNAs for HPV16 E6 oncogene have an effect on the E6/E7 bicistron *in vitro* as well as *in vivo*. Another group has reported the use of synthetic siRNAs for HPV18 E6 oncogene [74]. In this study, the induction of apoptosis of the CaSki cells (HPV16+), the increase of p53 and p21-CIP1/WAF1 expression, and the expression of the hypophosphorylated isoform of pRb were demonstrated. A finding that was noteworthy in this study was that siRNAs for HPV18 E6 did not affect HPV18 E7 expression. Initially, this observation seems to contradict what was reported by Jiang et al., who demonstrated that siRNAs for E6 oncogen have an effect on both E6 and E7 oncogenes [72]. Nevertheless, these data are not totally contradictory since they can be explained by the design of siRNA nucleotidic sequences. When we did a more in-depth analysis of both studies, we found that the design of siRNAs is directed to different sequences of E6 oncogene. This suggests a silencing effect that is dependent on the complementary position of the bases between siRNAs and mRNA. Therefore, the silencing of the HPV E6-E7 bicistronic transcript is dependent on the design of siRNA sequences for HPV E6 and E7 oncogenes. In addition, when analyzing the effect of HPV18 E6-E7 bicistron silencing, it was seen that administration of siRNAs specific for E7 induces silencing of both E6/E7 oncogenes, whereas siRNAs for E6 only inhibit E6 expression but do not have effects on E7 expression [75]. In the analysis of the design of siRNA sequences for E7, we observed that the complementarity of bases with the corresponding mRNA occurs in a position that affects the expression of the E6-E7 bicistron; nevertheless, siRNAs for E6 are complementary with mRNA in a sequence that does not influence the silencing of the E6-E7 bicistronic transcript. Again, this evidence supports the fact that the silencing of HPV E6-E7 bicistron expression is dependent on the design of the siRNA sequences and suggests that the alternative splicing of HPV E6/E7 oncogenes precedes the silencing by siRNAs. Additionally, this same study analyzed the functionality of siRNAs for E6/E7 and demonstrated the induction of expression in p53, p16, p21, p27, and in the hypophosphorylated isoform of pRb, the silencing of cyclin A gene, and the induction of apoptosis in human cancer cells. This evidence supports the fact that viral oncoproteins can have antiapoptotic properties by their influence on p53 protein function and when these oncogenes are silenced with siRNAs, p53 recovers the cellular cycle control functions and apoptosis [76]. Thus, E6 oncoprotein may have an important impact on other components of the apoptosis regulatory machinery. The high risk HPV E6 oncoproteins induce the proteolytic inactivation of certain proapoptotic proteins such as p53 [77], Bak [78], FADD [79], procaspase-8 [80], or c-Myc [81, 82]. In human keratinocytes immortalized by E6, low levels of apoptosis as compared to the non-immortalized control cells were observed after CD95 (Fas) agonist treatment [79]. Interestingly, in addition to p53 and p21, protein levels of antiapoptotic proteins Bcl-2 and Flip were reduced. Proteosomal inhibition increased the susceptibility of E6 expressing cells to CD95-mediated apoptosis. In addition, several studies have examined the sensitivity of cells expressing E6 to TNF. HPV-16 E6 was shown to bind to the C-terminal end TNF receptor 1 (TNF R1) and protect cells from TNF-induced apoptosis in mouse fibroblasts and human histiocyte/monocyte and osteosarcoma cells [83, 84]. E6 binding to TNF R1 interfered with the Fas pathway. Furthermore, use of an inducible E6 expression system demonstrated that this protection is dose dependent, with higher levels of E6 leading to greater protection. Although E6 suppresses activation of both caspase 3 and caspase 8, it does not affect apoptotic signaling through the mitochondrial pathway. Mammalian two hybrid are demonstrated that E6 binds directly to the death effectors domain of Fas-associated death domain (FADD) and to protect cells from Fas-induced apoptosis. In addition, binding to E6 leads to degradation of FADD, with the loss of cellular FADD proportional to the amount of E6 expressed. These results support a model in which E6-mediated degradation of FADD prevents transmission of apoptotic signals via the Fas pathway [85].

### 3.6. Chemotherapeutical Agents and siRNAs for HPV E6 and E7

Although we know the effect of different chemotherapeutical drugs on the expression of p53 protein in human tumoral cervical cells, we do not know if there is an association between the activation of gene p53, the cytotoxic effect of drugs, and the silencing of HPV oncogenes with siRNAs. Thus, different groups have analyzed the expression of p53 protein in HeLa cells (HPV18+) by the administration of siRNAs for the HPV18 E6 oncogene, combined with treatment by carboplatin, cisplatin, doxorubicin, etoposide, gemcitabine, mitomicine, mitoxantrone, oxaliplatin, paclitaxel, and topotecan [76]. In this study, the silencing of HPV18 E6/E7 oncogenes was observed, as well as an increase in p53 protein expression and changes in cytotoxicity that were dependent on the nature of each chemotherapeutical compound. Also, another group demonstrated, using HeLa cells, that the administration of specific siRNAs for HPV18 E6 generated in lentivirus, combined with cisplatin, the drug most frequently used in the treatment of advanced CC, produces the silencing of HPV18 E6 and E7 oncogenes, an increase in p53 expression and death of cancer cells by cellular senescence [86]. This evidence suggests that the silencing of HPV E6/E7 oncogenes by siRNAs can increase the cells sensitivity to the cytotoxic effects of drugs and that the combined treatment may have a synergistic effect in decreasing the resistance to chemotherapeutical drugs, which is an advantage for treatment.

### 3.7. siRNAs Transporting Molecules for HPV E6/E7

In the evaluation of the biological effects of siRNAs, *in vitro *as well as *in vivo, *protocols for the administration of synthetic siRNAs have been developed, for HPV E6/E7 oncogenes, where these molecules are coupled with liposomes as transport vehicles [87]. In these studies, it has been shown in CaSki (HPV16+) cells that when siRNAs for HPV16 E6/E7 are administered, silencing of both oncogenes is induced and the cancer cells die by apoptosis. With this same system, and through the development of a murine tumor model with CaSki cells, the effects of siRNAs for E6 have been evaluated and the silencing of the viral oncogene has been determined, as well as the induction of apoptosis of tumor cells and a significant inhibition of the growth of the tumoral mass *in vivo *[87]. Although these findings are significant, when synthetic siRNAs are used and administered by lipofection to mammalian cells, a potential problem that appears is the cleavage of siRNAs by the action of endogenous cellular endonucleases. An alternative design to protect the siRNAs from this cleavage is the synthesis of siRNAs with chemical modifications; however, this may induce undesirable collateral effects. Another problem that arises in the systemic administration of siRNAs is that we do not have a dose dependent effect on target organs. Thus, in order to overcome these methodological inconveniences, it has been reported that the specific siRNAs for HPV oncogenes may be administered in a liposome based system contained in biogels [88]. The combination of adhesive biogels and liposomes containing siRNAs for the HPV16 E7 oncogene has resulted in the specific silencing of E7 and the induction of apoptosis of cancerous cells *in vitro.* In addition, the use of atelocollagen as a vehicle to administer the siRNAs for HPV18 E6 and E7,* in vitro *as well as *in vivo, *has also been reported [89]. In this study, it has been observed that siRNAs silence E6/E7 oncogenes expression, inhibit cellular proliferation, induce the expression of the hypophosphorylated isoform of pRB, and induce death of cancerous cells by cellular senescence. In this same system, it was also demonstrated that the administration of siRNAs for HPV18 E6/E7, attached to atelocollagen, inhibits the growth of the tumor mass in a murine tumoral model [89]. Although the silencing effects of synthetic siRNAs are evident, the half life of these molecules after their administration is relatively short, even when they are attached to transporting molecules; this limits their application in preclinical or clinical trials. Besides this, the real application of siRNAs for high-risk HPV oncogenes in clinical phase studies, requires a better understanding of the development of highly specific siRNAs and greater efficiency in the *in vivo *liberation systems. In this sense, protocols have been developed for the generation of lentiviruses as molecular liberation vectors for specific siRNAs for high-risk HPV E6 and E7 oncogenes, as well as for their stable transfection and transduction in human CC cells. The biological effects of silencing with siRNAs, *in vitro *as well as *in vivo *models, are being evaluated [90]. 

### 3.8. Transcriptome Regulation by the Effect of siRNAs for HPV E6/E7

Another aspect of the silencing of HPV E6/E7 oncogenes that has been studied, using siRNAs, is the effect it has on transcriptome regulation of human tumor cervical cells. In 2007, Kuner et al. [91] analyzed the transcriptome of HeLa cells and cervical cells from patient biopsies, inducing the silencing of HPV 18 E6 and E7 with siRNAs generated in the silencing plasmid of pSUPER. In this study, 360 cellular genes were identified which had a negative regulation and 288 genes with positive regulation due to the effect of siRNAs for E6/E7. Most of these genes are involved in relevant biological processes during the development of the tumor cell, such as: apoptosis control, regulation of the cell cycle, formation of the mitotic spindle, processing of mRNA by splicing, metabolism, DNA replication and repair, nuclear transport, cell proliferation, and gene regulation by c-Myc. These findings complement previous studies where the expression of HPV E2 protein has been analyzed. This protein inhibits HPV E6/E7 expression, and the expression of the transcriptome in human tumor cervical cells. The potential of this type of studies lies in the fact that the basic cell pathways for viral transformation may be identified, which may be targets for the development of therapeutical strategies. Also, new molecular biomarkers may be found for diagnosis and prognosis of CC. An example of these biomarkers is the enhancer of zeste homolog 2 (EZH2), which is repressed by the inhibition of E6/E7 by siRNAs. These data suggest that this biomarker is active in CC cells transformed by HPV [91]. Thus, the information generated by the study of the transcriptome of CC cells, using siRNAs for HPV oncogenes, may contribute to the diagnosis prognosis and treatment of this neoplasia. It has been previously demonstrated that silencing E6 and E7 has effects on several cellular targets. For example, a connection between oncogenic HPVs and C-MYC during the transformation process has been previously discussed. For example, insertion of HPVs close to the MYC locus is observed in about 10% of HPV positive genital cancers, and it has been speculated that the HPV transcriptional control region may induce overexpression of the nearby MYC gene [92]. Ectopically expressed E6 has been reported to stimulate the C-MYC promoter, and E6 and/or E7 can increase C-MYC expression under certain experimental conditions [93]. The data on transcriptome regulation by siRNA, for E6 and E7, would be consistent with the idea that the viral E6/E7 genes can either directly or indirectly activate C-MYC expression; this may be an alternative mechanism for inducing downstream C-MYC targets during HPV-associated carcinogenesis.

## 4. Conclusions 

Cervical lesions caused by HPV persistent infection can be removed naturally by the host immune response, and only a small percentage may progress to cancer, thus, the immune response is essential for the control of precursor lesions and CC. Therefore, to know the molecular mechanisms and potential targets for blocking HPV-induced, lesion development is of high relevance, because 80% percent of CC cases are in developing countries. HPV prophylactic vaccines and construction of the sanitary structure are very expensive; therefore, to develop techniques base on genomic approach will be very useful for CC therapy. The relevance of this approach will be better appreciated once it is applied in clinical protocols. 

The correction of immune-activating signals, eradication of inhibitory factors, and the evasion from newly developed immunoresistant tumor phenotypes need to be simultaneously considered.

## Figures and Tables

**Table 1:** 

HPV oncogenes	siRNA design	Biological effects	References
HPV16 E6 and E7	Synthetic siRNA	Silencing of HPV16 E6 and E7, p53, p21 and pRb expression. Apoptosis induction *in vitro*.	[72]
HPV16 E6	Synthetic siRNA	Silencing of HPV16 E and E7, p53, p21 and pRb expression. Cellular proliferation inhibition *in vitro*. Tumor growth inhibition *in vivo*.	[73]
HPV18 E6	Synthetic siRNA	Silencing of HPV16 E6, p53, p21, and pRb expression. Apoptosis induction *in vitro*.	[74]
HPV16 E7	Synthetic siRNA in bio-adhesive gels	Silencing of HPV16 E7. Apoptosis induction *in vitro*.	[87]
HPV18 E6 and E7	Synthetic siRNA with chemotherapy	Silencing of HPV18 E6 and E7, as well as p53 expression. Cytotoxicity decrease *in vitro*.	[94]
HPV18 E6	siRNA in lentivirus with chemotherapy	Silencing of HPV18 E6 and E7, as well as p53 expression. Cytotoxicity decrease *in vitro*.	[86]
HPV16 E6 and E7	Synthetic siRNA in liposomes	Silencing of HPV16 E6 and E7. Apoptosis induction *in vitro*. Tumor growth inhibition *in vivo*.	[88]
HPV18 E6 and E7	siRNA with atelocollagen	Silencing of HPV18 E6 and E7, as well as p53 and pRb expression. Cellular senescence induction *in vitro*. Tumor growth inhibition *in vivo*.	[89]
HPV18 E6 and E7	Synthetic siRNA	Silencing of HPV18 E6 and E7 and cyclin A gene, as well as p53, pRb, p16, p21, and p27 expression. Apoptosis induction *in vitro*.	[75]
HPV18 E6 and E7	siRNA in pSUPER plasmid	Silencing of HPV16 E6 and E7, as well as p53 and pRb expression. Transcriptome expression analysis of cancer cells.	[91]
HPV16 E6 and E7	siRNA in psiCheck 2 plasmid	Silencing of HPV16 E6 and E7, as well as p53 and p21 expression. Cellular senescence induction *in vitro*. Tumor growth inhibition *in vivo. *	[95]
HPV16 E7	siRNA in pSIRE-DNR plasmid	Silencing of HPV E6 and E7, as well as p53, p21 and pRb expression. Apoptosis induction *in vitro*.	[96]
High risk HPV E6 and E7	siRNA in lentivirus	Silencing of HPV16 E6 and E7. p53 and p21 expression. Apoptosis induction *in vitro* and *in vivo*. High efficiency of infection of proliferation cells and quiescent cells.	[90]
HPV16 E6 and E7	siRNA in pSilencer 1.0U6-plasmid	Silencing of HPV16 E6 and E7. p53 and pRb expression. Cellular proliferation inhibition. Autophagic and apoptosis induction of tumor cells *in vivo*.	[97]
